# Lung Ultrasound Guided Fluid Management Protocol for the Critically Ill Patient: study protocol for a multi-centre randomized controlled trial

**DOI:** 10.1186/s13063-019-3345-0

**Published:** 2019-04-25

**Authors:** Daniel-Mihai Rusu, Ianis Siriopol, Ioana Grigoras, Mihaela Blaj, Adi-Ionut Ciumanghel, Dimitrie Siriopol, Ionut Nistor, Mihai Onofriescu, Gigel Sandu, Beatrice Cobzaru, Dragos Viorel Scripcariu, Olguta Diaconu, Adrian Constantin Covic

**Affiliations:** 1 Grigore T. Popa University of Medicine and Pharmacy, Str. Universității nr. 16, 700115 Iasi, Romania; 2 Anaesthesia and Intensive Care Department, Regional Institute of Oncology, Str. General Henri Mathias Berthelot 2-4, 700483 Iasi, Romania; 3 Anaesthesia and Intensive Care Department, Saint Spiridon University Hospital, Iasi, Romania; 4 Nephrology Department, Dr. C.I. Parhon University Hospital, Iasi, Romania; 5 Surgery Department, Regional Institute of Oncology, Iasi, Romania

**Keywords:** Lung ultrasonography, B-lines score, Fluid management, Intensive care, Critically ill patient, Randomized controlled trial

## Abstract

**Background:**

In routine intensive care unit (ICU) practice, fluids are often administered without a safety limit, which may lead to fluid overload and decreased survival. Recently, B-lines score (BLS) has been validated as a lung ultrasound (LUS) quantification of pulmonary congestion. This suggests that LUS may provide a safety threshold to conduct fluid therapy and to avoid overhydration. However, there is no randomized study to test the utility of LUS in guiding fluid management in ICU patients by using a pre-specified BLS cut-off value as a threshold for fluid removal.

**Methods:**

LUS Guided Fluid Management Protocol for the Critically Ill Patient is a prospective, multi-centre, randomized controlled trial. Five hundred ICU patients will be randomly assigned in a 1:1 ratio, to protocolized LUS-based fluid management or usual care. The trial intervention will start on ICU admission and will consist in daily assessment of BLS and triggered evacuation of excessive fluids with loop diuretics (Furosemide) when BLS ≥ 15. If rebalancing volume status with diuretics fails, forced evacuation by ultrafiltration will be used. The main endpoint is death from all causes at 28 days from randomization. The secondary outcomes are presence and time-course evolution of organ dysfunctions, ICU- and hospital length of stay, all-cause mortality at 90 days, and health economics data.

**Discussion:**

If study results will show that LUS guided fluid management protocol improves outcome in ICU patients, it will be the base for other studies to refine this protocol or track those categories of critically ill patients to whom it may bring maximum benefits.

**Trial registration:**

ClinicalTrials.gov, NCT03393065. Registered on 8 January 2018.

**Electronic supplementary material:**

The online version of this article (10.1186/s13063-019-3345-0) contains supplementary material, which is available to authorized users.

## Background

In critically ill patients, fluids are used to optimize organ perfusion, compensate fluid losses, avoid fluid deficiency, and deliver medication, antibiotics, or nutrition. However, when in excess, fluids may induce organ dysfunctions [1–3], prolong intensive care unit (ICU) and hospital length of stay [4, 5], and even decrease survival [2–11]. Despite evidence regarding harmful effects of overhydration, in routine ICU practice, fluids are often administered without a safety limit [12] and diuretics are frequently prescribed without an agreement upon what would be the targeted endpoint [13]. These practices may be attributed to the drawbacks of currently used volume assessment methods.

In recent years, LUS has emerge as a novel tool to assess overhydration and has been successfully used in patients from the nephrology [14–19], cardiology [20], and ICU departments [21–24]. LUS has the advantages of being safe, non-invasive, rapidly available, and already part of different diagnostic algorithms for life-threatening conditions [25–27]. Being able to detect in real time the increase of extravascular lung water (EVLW) [28, 29], as well as the response to excessive fluids removal [19, 20], LUS may provide a valuable safety threshold to conduct fluid therapy and to optimize volume status.

The sonographic signs of increased EVLW are the artefacts called B-lines [24, 30–37]. B-lines are hyperechoic, comet-tail artefacts, which emerge from the level of the pleural line and move synchronously with lung sliding [38, 39]. The correlation between B-lines and the amount of EVLW has been demonstrated, by comparing LUS with gold standard methods for EVLW assessment [21, 24, 32]. B-lines presence may be evaluated by scanning the chest, from the second to the fourth intercostal space on the left side, and from the second to the fifth intercostal space on the right side, at parasternal, mid-clavicular, anterior-axillary, and mid-axillary lines, with the patient in supine position [35, 36]. The sum of all B-lines yields a score, B-lines score (BLS), which assess the degree of lung congestion [24, 39]. A BLS < 5 is considered normal, while a BLS > 15 reflects moderate/severe pulmonary congestion [39].

Some prospective studies have shown that a high BLS is linked to a higher risk of mortality in dialysis patients [17, 40] and heart failure patients [41–45]. Few prospective studies have also used LUS as a prognostic tool in critically ill patients [22, 46]. However, there is no randomized study to test the utility of LUS in guiding fluid management in ICU patients, by using a pre-specified BLS cut-off value, as a threshold for fluid removal.

## Study hypothesis

Starting from the premises that mortality may be decreased by avoiding fluid overload and LUS may detect overhydration in early stages, we believe that daily evaluation of BLS, as a refined LUS quantification of pulmonary congestion and triggered evacuation of excessive fluids with diuretics or by ultrafiltration when BLS ≥ 15, may improve outcome in critically ill patients.

Secondary hypotheses are that this intervention is cost-effective and may prevent or, if already present, may improve time-course evolution of organ dysfunctions, may reduce the ICU and hospital length of stay, and increase 90-day survival.

## Objectives

The aim of this study is to evaluate a fluid management protocol for adult ICU patients, based on the daily assessment of BLS using LUS, compared to usual care. A pre-specified BLS cut-off value of 15 will be used in this randomized study to correct fluid overload. The selected cut-off value is recommended by Picano et al. as the limit between absent/mild to moderate/severe lung congestion [39].

## Methods/Design

### Study design

LUS Guided Fluid Management Protocol for the Critically Ill Patient is a multi-centre, randomized controlled two-arm trial, with 1:1 allocation of ICU patients to LUS guided fluid management or usual care.

While hospitalized in the ICU, participants will be randomly assigned to receive LUS guided fluid management or usual care, until ICU discharge or for up to 28 days after randomization, whichever comes first.

The main endpoint will be all-cause mortality at 28 days from randomization (ICU admission).

**Fig. 1:**
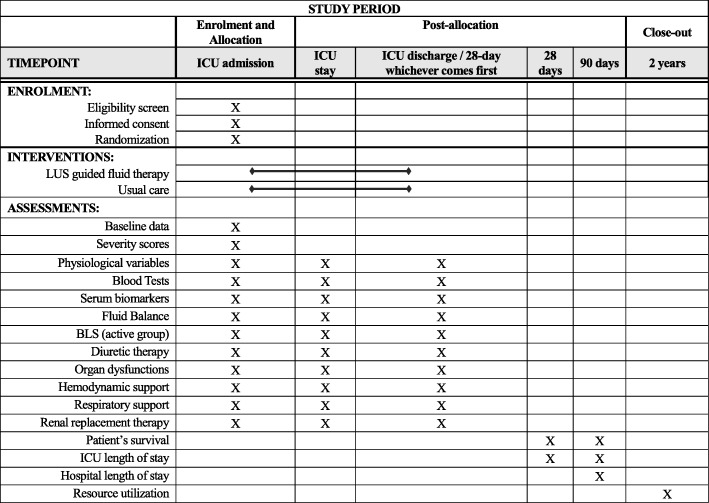
The schedule of study procedures (SPIRIT figure)

The Standard Protocol Items: Recommendations for Interventional Trials (SPIRIT) checklist is provided in Additional file 1 and the schedule of study procedures (SPIRIT figure) is shown in Fig. 1.

### Settings

The trial will be conducted in the ICUs of two university hospitals: Saint Spiridon University Hospital and Regional Institute of Oncology Iasi. The ICUs involved in the trial follow the recommendations of the Society of Critical Care Medicine regarding ICU admission and discharge criteria [47], can deliver the same high level of care, and have physicians capable to provide LUS guided fluid therapy. A rapid response team exists in each of these hospitals and ensures on-demand, hospital ward guidance for the management of patients with deteriorating conditions, as well as the evaluation of patients that may benefit from ICU admission.

### Participants

We aim to enroll 500 adult ICU patients over a period of 24 months. To identify eligible individuals, a daily screening of all critically care admissions will be performed by study investigators. Patients will be included in this study if they are expected to stay at least 48 h in the ICU, meet at least one of the following inclusion criteria, and have none of the exclusion criteria.

#### Inclusion criteria

1. Major surgery
2. Major co-morbidities in surgical patients
3. Polytrauma with Injury Severity Score (ISS) ≥ 15
4. Acute Physiology and Chronic Health Evaluation II Score (APACHE II) on admission ≥ 10
5. Sequential Organ Failure Assessment Score (SOFA) on admission ≥ 6

#### Exclusion criteria

1. Patient’s refusal
2. Age < 18 years
3. Pregnancy
4. Patient with known pulmonary conditions that interfere with the interpretation of LUS: pneumectomy; pulmonary fibrosis; persistent pleural effusion
5. Stage 5 chronic kidney disease or indication for emergency renal replacement therapy (RRT)
6. Prolonged resuscitation (≥ 10 min) for cardiorespiratory arrest

### Ethical aspects

The study was approved by the Research Ethics Committee of the Grigore T. Popa University of Medicine and Pharmacy Iasi (date 14 November 2017, number 26261) and by the Research Ethics Committees of each hospital involved in the trial. Patients will receive written information about the nature of the trial, its aims and expected advantages, as well as possible risks, and they will be asked to sign an informed consent. If a patient is unable to give consent at the time of ICU admission, a legally authorized representative may give authorization. Once the participant regains capacity, the individual will be asked to confirm or withdraw consent.

### Randomization and blinding

Study patients will be randomly assigned to either LUS guided fluid management or usual care, in a 1:1 ratio, using block randomization. The randomized block design will be created using a computer-based program by a study team member not involved in patient enrollment and treatment. This team member will securely guard the randomized block design at the coordinating center and will provide the allocation sequence each time a new patient is enrolled. Due to study design, patients and ICU physicians cannot be blinded, but the researcher that investigates primary and secondary outcomes will be blinded to patient group assignment.

### Study interventions

#### LUS guided fluid management (active group)

LUS guided fluid management is based on BLS assessment using LUS, within the first 24 h of ICU admission, and daily thereafter, until ICU discharge, or for up to 28 days after randomization, whichever comes first.

After the initial LUS examination, the follow-up LUS will be performed daily, in the morning, at set times, between 9 am and 11 am. The rationale for this approach is that all ICU patients will have daily, in the morning, a full clinical examination and a complete set of laboratory tests, providing evaluation of fluid status. In this way, all collected data may be further correlated with LUS findings.

LUS examinations will be performed at bedside, with the patient in a supine position, using the 28 zone technique and the GE LOGIQ V2® ultrasound system with the GE 3Sc-RS Cardiac Sector Probe® (1.5–4.0 MHz frequency). The focus of the image will be set at the level of the pleural line and the depth of penetration at around 4–8 cm. The image will be optimized by regulating the gain. The lungs will be scanned, from the second to the fourth intercostal space on the left side, and from the second to the fifth intercostal space on the right side, at parasternal, mid-clavicular, anterior-axillary, and mid-axillary lines. B-lines will be recorded in each intercostal space (28 sites of examination). The sum of all B-lines will produce a score, BLS, reflecting the extent of EVLW accumulation.

The ICU physician may recommend fluid therapy or vasoactive drugs with types and amounts at his/her choice. However, when signs of moderate/severe pulmonary congestion are seen on LUS exam (BLS ≥ 15), a negative 24-h fluid balance (250–1000 mL) will be intended according to clinical judgement.

To achieve this goal, a protocolized intravenous administration of furosemide will be used, under careful monitoring of diuretic response. Diuretic administration will start with a furosemide dose ≤ 80 mg/day which will be further adapted based on the next 24-h fluid balance, the follow-up BLS, and the previous diuretic regimen. If a negative fluid balance of > 1000 mL/24 h is obtained and BLS is still ≥ 15, the initial dose of diuretic will be reduced. If a negative fluid balance of < 1000 mL/24 h is obtained and BLS is still ≥ 15, the administered diuretic dose will be maintained. If negative fluid balance is not achieved and BLS is still ≥ 15, the dose of diuretic will progressively be increased until the goal is attained. The maximum dose of furosemide will not exceed 800 mg/day.

In case of a fall in blood pressure or an increase in creatinine blood level that is perceived to be due to a transient episode of intravascular fluid depletion, the diuretic may be temporarily stopped or the dose may be decreased according to clinical judgement. After the patient has stabilized and if BLS is ≥ 15, diuretics will be reinitiated until the patient’s BLS is < 15. If the target of depleting overhydrated patients with diuretics cannot be achieved, RRT will be used. For RRT, the treating physician may choose between slow continuous ultrafiltration, continuous veno-venous hemofiltration, continuous veno-venous hemodialysis, or continuous veno-venous hemodiafiltration. Along with the goal of rebalancing fluid status, the choice of RRT technique should rely on patients’ particularities and other goals of homeostasis restoration.

As acute kidney injury (AKI) is often present in ICU patients, and RRT is a common procedure during critical care, to avoid bias, AKI will be diagnosed in all patients based on changes in the serum creatinine, urine output, or both, according to the KDIGO recommendations [48].

In AKI patients, the following criteria for RRT initiation will be used:

1. Stage 3 AKI (urine output < 0.3 mL/kg/h for ≥ 24 h and/or > 3-fold increase in serum creatinine level compared with baseline, or serum creatinine of ≥ 4 mg/dL with an acute increase of at least 0.5 mg/dL within 48 h)
2. Urine production < 200 mL/12 h or anuria
3. Serum potassium level > 6 mEq/L and/or with electrocardiographic abnormalities
4. Organ edema in the presence of AKI resistant to diuretic treatment
5. A pH < 7.15 in the context of either pure metabolic acidosis or mixed acidosis
6. Acute pulmonary edema due to fluid overload responsible for severe hypoxemia despite diuretic therapy

**Fig. 2:**
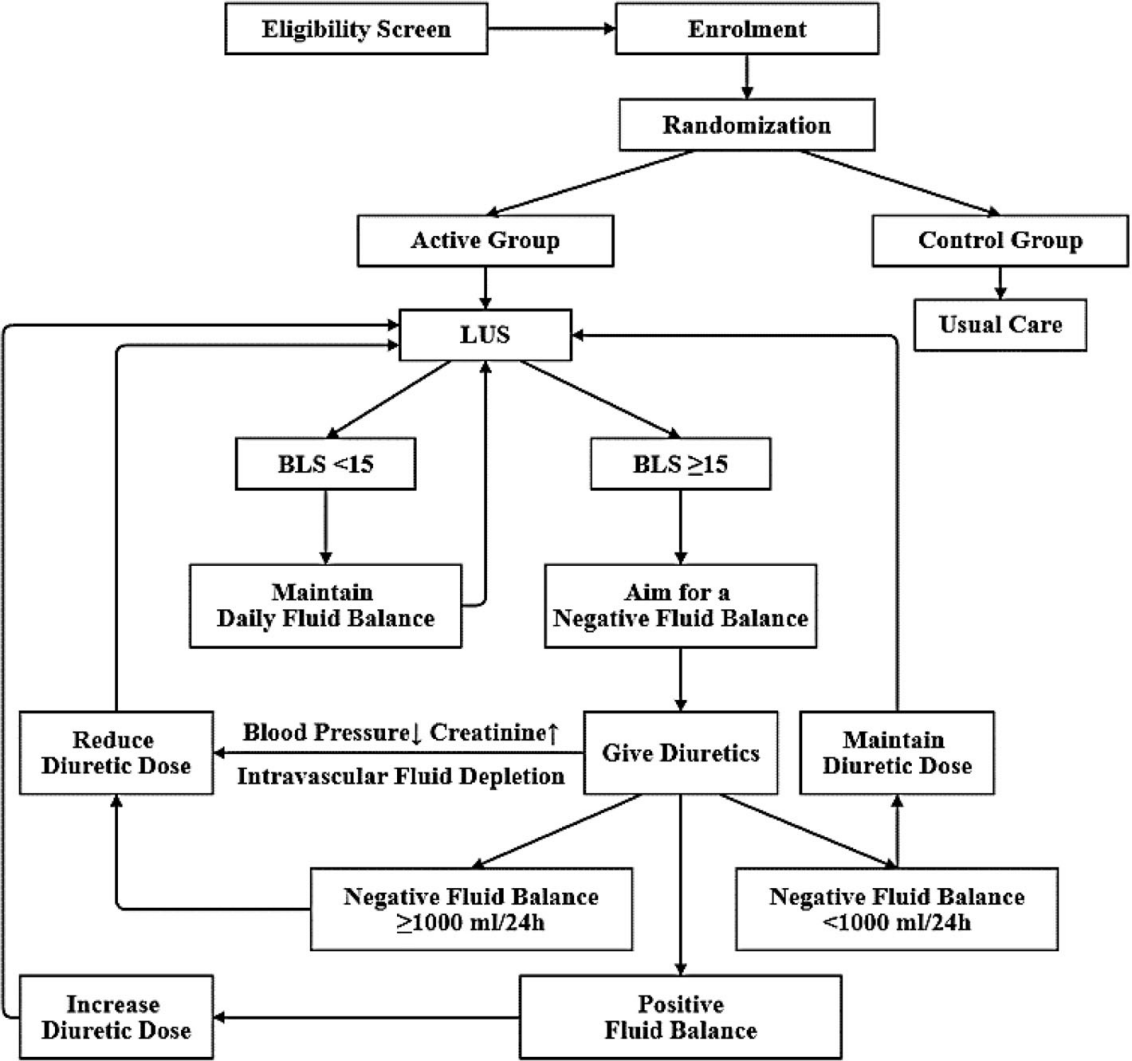
Algorithm of study protocol

The algorithm of study protocol is shown in Fig. 2 and the recommended diuretic regimen in patients with BLS ≥ 15 is shown in Table 1.

**Table 1:** Recommended intravenous diuretic regimen in patients with BLS ≥ 15

	Furosemide (mg/day)	
	Previous daily dose	Recommended daily dose
1	≤ 80	40 mg i.v. bolus + 5 mg/h
2	81–160	80 mg i.v. bolus + 10 mg/h
3	161–240	80 mg i.v. bolus + 20 mg/h
4	> 240	80 mg i.v. bolus + 30 mg/h

#### Usual care (control group)

Usual care is defined as fluid and diuretics administration according to the clinical judgement of the treating ICU physician. The prescribed fluid’s type and amount, as well as the dose of diuretics, will be at the discretion of the ICU physician, guided by clinical and laboratory data, except LUS.

### Data collection and follow-up

Data will be collected on paper-based Case Report Forms and in a secure electronic database for further analysis.

#### Clinical data

Baseline data will include patients’ age, gender, weight and height, reason of ICU admission, data on acute conditions and co-morbidities, on recent immunosuppressive treatments, inflammatory status, infectious status, and severity scores (APACHE II, SOFA, and ISS [only for polytrauma patients]). Except for SOFA severity score, which will be recorded daily, all other severity scores will be calculated once, at ICU admission.

Physiological variables (temperature, heart rate, blood pressure, cardiac rhythm, peripheral blood oxygen saturation, presence of pulmonary crackles or edema), as well as routine laboratory tests (complete blood count, serum electrolytes, urea, and creatinine), will be recorded daily, during ICU stay. Based on case complexity and evolution, all other needed monitoring parameters (e.g. invasive hemodynamic monitoring parameters) and laboratory tests (e.g. arterial-blood gas tests, central venous oxygen saturation, lactate level, inflammatory and infectious biomarkers) will be recorded.

#### Biomarkers

Blood samples will be collected and stored from all study patients, regardless of group allocation, for biomarkers measurement. Serum brain natriuretic peptide (NT-proBNP), interleukin-6 (IL-6), and neutrophil gelatinase-associated lipocalin (NGAL) will be assessed by electro-chemiluminescence “sandwich” immunoassays, using the Roche Elecsys® kit, based on polyclonal antibodies against NT-proBNP, IL-6, and NGAL, respectively. Blood samples will be collected on ICU admission, on the third, fifth, and seventh day of ICU stay, and weekly thereafter, until ICU discharge.

#### Fluid balance and diuretics regimen

Detailed data about daily fluid balance, including route, type and amount of fluid solutions received, and type and volume of fluid losses, as well as data upon diuretic therapy, will be collected daily, during ICU stay.

BLS will be recorded daily in the active group only.

#### Outcome data

Presence and time-course evolution of organ dysfunctions as well as specific therapeutic interventions will be recorded accordantly: hemodynamic support (drug type, dose, and duration of treatment); respiratory support (number of hours on mechanical ventilation); and RRT (type, duration, dose of dialysis, volume of removed fluid, pre and post dialysis BLS in active group only). All complications related to the RRT use will be recorded.

Survival/non-survival at 28 and 90 days since randomization.

### Primary outcome

The main outcome will be death from all causes at 28 days from randomization. This will be assessed by a study team researcher, blinded to patient group assignment, based on hospital recordings or a brief telephone interview with the patient or patients’ legally authorized representative or relative.

### Secondary outcomes

Secondary outcomes will include: presence and time-course evolution of organ dysfunctions during ICU stay; changes in SOFA severity score and biomarkers (NT-proBNP, IL-6, NGAL); ICU length of stay; total hospital length of stay; all-cause mortality at 90 days; resource utilization; and cost–benefit ratio of used fluid solutions (crystalloids/colloids). Secondary outcomes will be assessed based on recorded data during ICU stay, hospital files, and a short telephone interview with the patient or patients’ legally authorized representative to establish 90 days’ survival.

### Strategies to ensure adequate enrolment and protocol compliance

Case Report Forms will be uploaded into a secure database at the trial coordination center in a timely manner. This will allow periodical checks for enrolment rates, data accuracy, and protocol compliance. Whenever necessary, trial coordination center will provide support and feedback to the site investigators.

A safety interim analysis will be conducted after the enrolment of the first 100 patients.

### Statistical analysis

Statistical analysis will be performed using SPSS (SPSS Inc., Chicago, IL, USA) and conducted on an intention-to-treat basis. Frequency distributions will be calculated for all variables and descriptive statistics will be used to define patient’s characteristics in both arms. Study groups will be compared using non-parametric and parametric statistics, according to the types of variables analyzed.

The relative risk of 28-day mortality, with 95% confidence interval, will be assessed in both trial arms. Based on identified mortality-associated variables, we will also perform sensitivity analyses, subgroups analyses, and interactions tests.

Secondary outcomes will be analyzed using univariate analysis and competing-risk methods.

### Sample size power calculation

Based on sample size power calculations, for a targeted power of 80% and alpha value of 0.05, the number of patients needed to reveal a significant decrease of 10% in mortality is about 500 (250 in each arm). The mortality benefit estimation is based on a large cohort study of ICU patients, which showed that without any specific intervention, the presence/absence of fluid overload resulted in a mortality rate of 20% and 16.8%, respectively [4].

### Strengths and limitations of the study protocol

To our knowledge, this is the first prospective randomized controlled study that aims to evaluate a fluid management protocol for adult ICU patients, based on the daily assessment of BLS compared to usual care. A pre-specified BLS cut-off value of 15 will be used to assess fluid overload.

Our study will address both surgical and medical ICU patients. To identify eligible patients, we use severity scoring systems, but inclusion criteria are not limited to them, as scoring systems alone may be poor determinants of the level of care. ICU patients at risk for postsurgical organ dysfunction due to surgery magnitude or co-morbidities are also included.

LUS data may depend on examiner, ultrasound system, and patients’ particularities. In order to minimize bias, LUS will be performed by previously trained investigators, using the same type of ultrasound equipment, probe, and technique. Patients with pulmonary conditions known to interfere with BLS evaluation (pneumectomy, pulmonary fibrosis, persistent pleural effusion) will be excluded, but other conditions, such as obesity, extrapulmonary diseases in the examination area, cardiac assist devices, or wound dressing, may also render the LUS examination difficult. To monitor LUS efficiency, B-lines will be counted in every site of examination and inaccessible sites for B-lines visualization will be noted. Although we cannot exclude all bias, the before mentioned strategies aim to minimize potential errors.

Diuretic prescription in overhydrated hemodynamically unstable patients may result in decreased protocol adherence. Therefore, protocol deviations (e.g. deviation from diuretic regimen) will be recorded and analyzed.

Due to the study design, the ICU physician in charge is not blinded to each patient’s group allocation. Investigators in charge with biomarkers measurement and outcome assessment will be blinded. This may influence fluids and diuretics prescription, which may further modify the incidence of fluid overload and alter the results.

## Discussion

The attempt to improve fluid management in critically ill patients has led in the last few years to a refined approach, centred on volemic status and fluid responsiveness assessment. Many studies have addressed the hemodynamically unstable patient and assessed different methods used to predict fluid responsiveness [49]. Less attention has been given to the diagnosis and management of fluid overload, mainly in patients at risk for organ dysfunction. Our study protocol includes patients at risk (patients after major surgery, surgical patients with major co-morbidities) and concentrates on active measures to detect and correct fluid overload.

Dynamic measurements of cardiac output and related parameters, although proven to be superior to static methods in directing fluid therapy [49], are still marginally used in clinical practice [50]. Offering fast results, being safe, non-invasive, and easy to learn [39, 51, 52], LUS has the potential to overcome the drawbacks of other fluid assessments methods. If our study hypothesis proves to be right, LUS and BLS may help to discriminate between patients that may benefit from further fluid repletion and patients in which a fluid evacuation strategy should be adopted. LUS is not supposed to replace invasive hemodynamic monitoring or other methods used to assess fluid responsiveness, but to help intensive care physicians by providing a safety threshold for fluid resuscitation. More than this, for patients with pulmonary congestion, an algorithm for fluid management is proposed, with specific therapeutic measures that should be taken in order to achieve specific endpoints.

Study results will be submitted to an ISI indexed scientific journal. Other dissemination methods will include presentations at national and international scientific meetings. Favourable results may also be the ground for further research aiming for fluid management protocols based on non-invasive, simple, reliable, bedside techniques.

## Trial status

Patient enrollment in the trial started in November 2017 and is expected to end in October 2019. The trial is registered at ClinicalTrials.gov, NCT03393065. Registered on 8 January 2018 – retrospectively registered. Protocol version: 1.

## Additional file

Additional file 1: SPIRIT Checklist. (DOCX 50 kb)

## Abbreviations

AKI: Acute kidney injury
APACHE II: Acute Physiology and Chronic Health Evaluation II Score
BLS: B-lines score
EVLW: Extravascular lung water
ICU: Intensive Care Unit
IL-6: Interleukin-6
ISS: Injury Severity Score
LUS: Lung ultrasound
NGAL: Neutrophil gelatinase-associated lipocalin
NT-proBNP: Brain natriuretic peptide
RRT: Renal replacement therapy
SOFA: Sequential Organ Failure Assessment Score
